# The movement-to-music (M2M) study: study protocol for a randomized controlled efficacy trial examining a rhythmic teleexercise intervention for people with physical disabilities

**DOI:** 10.1186/s13063-021-05751-2

**Published:** 2021-11-07

**Authors:** Hui-Ju Young, Byron Lai, Tapan Mehta, Mohanraj Thirumalai, Jereme Wilroy, Alex Yates, Brandon Kane, James H. Rimmer

**Affiliations:** 1 UAB/Lakeshore Research Collaborative, School of Health Professions, University of Alabama at Birmingham, Birmingham, AL USA; 2 Division of Pediatric and Rehabilitation Medicine, School of Medicine, University of Alabama at Birmingham, Birmingham, AL USA; 3 Department of Health Services Administration, School of Health Professions, University of Alabama at Birmingham, Birmingham, AL USA; 4 Department of Physical Medicine and Rehabilitation, School of Medicine, University of Alabama at Birmingham, Birmingham, AL USA

**Keywords:** Movement-to-music, Cardiorespiratory fitness, Mobility, Health, Disability, Randomized controlled trial

## Abstract

**Background:**

People with physical disabilities need exercise routines that are enjoyable, readily available in the home, adapted to their functional level, and eliminate common barriers to exercise participation related to transportation and time commitment. The purpose of the movement-to-music (M2M) study is to address these issues by establishing a remotely delivered, rhythmic exercise program for people with physical disabilities.

**Methods:**

The study is a two-arm randomized controlled efficacy trial examining a 12-week remotely delivered M2M intervention (eM2M) in 108 people with physical disabilities. The primary outcomes are changes in cardiorespiratory fitness and muscle strength at post 12-week intervention.

**Discussion:**

The eM2M study will enhance our understanding of an alternative intervention design and delivery mode that removes common barriers to exercise participation experienced by people with physical disabilities. The eM2M intervention may be an alternative option for people with physical disabilities to obtain regular exercise, especially during a pandemic when exercising in indoor facilities may be problematic.

**Trial registration:**

ClinicalTrials.gov NCT03797378. Registered on January 9, 2019, with the trial name “Movement-to-Music: Lakeshore Examination of Activity, Disability, and Exercise Response Study (M2M LEADERS)”.

## Administrative information

Note: the numbers in curly brackets in this protocol refer to SPIRIT checklist item numbers. The order of the items has been modified to group similar items (see http://www.equator-network.org/reporting-guidelines/spirit-2013-statement-defining-standard-protocol-items-for-clinical-trials/).

**Table:** 

Title {1}	The movement-to-music (M2M) study: study protocol for a randomized controlled efficacy trial evaluating an adaptive rhythmic teleexercise intervention for people with physical disabilities.
Trial registration {2a and 2b}.	ClinicalTrials.gov NCT03797378. Registered on January 9, 2019.
Protocol version {3}	Protocol Version 1; November 2020.
Funding {4}	National Institute on Disability, Independent Living, and Rehabilitation Research (NIDILRR). Grant number 90DPGE0005.
Author details {5a}	^1^UAB/Lakeshore Research Collaborative, School of Health Professions, University of Alabama at Birmingham, Birmingham, AL. ^2^Division of Pediatric and Rehabilitation Medicine, School of Medicine, University of Alabama at Birmingham, Birmingham, AL. ^3^Department of Health Services and Administration, School of Health Professions, University of Alabama at Birmingham, Birmingham, AL. ^4^Department of Physical Medicine and Rehabilitation, School of Medicine, University of Alabama at Birmingham, Birmingham, AL.
Name and contact information for the trial sponsor {5b}	Pimjai Sudsawad, ScD National Institute on Disability, Independent Living, and Rehabilitation Research (NIDILRR) Administration for Community Living (ACL) US Department of Health and Human Services (HHS) Office: 2212-B 330 C Street SW Washington, DC 20201 202-795-7447 Pimjai.sudsawad@acl.hhs.gov
Role of sponsor {5c}	The sponsor plays no part in study design, data collection, data management, data analysis, data interpretation, and decision to submit results for publication.

## Introduction

### Background and rationale {6a}

Exercise trials for people with physical disabilities are currently challenged by the inability to reach large, heterogenous groups of participants that are representative of the target population. Studies typically incorporate highly controlled designs and focus on examining one specific disability group in an exercise intervention with an average sample size of 40 participants per study [1, 2]. Because of this, the generalizability and transferability of the research is limited, which creates a need to identify evidence-based exercise interventions that are robust enough to be successfully used by multiple disability groups [3]. To address this limitation, one potential strategy is for studies to include people within the exercise intervention based upon their physical function, as opposed to a specific type of disability.

Creating exercise interventions for different levels of physical function present a few challenges. For example, when an exercise intervention is delivered to participants with various levels of functional mobility, people with higher or lower levels of physical function may perceive the program as not challenging or too difficult, which may compromise the physiological adaptations achieved from the intervention. Froehlich-Grobe et al. examined the effectiveness of a physical activity intervention for women with a wide variety of mobility limitations and reported no significant physiologic change [4]. They suggested that a possible reason for this nonsignificant finding was the variability in functional mobility levels. The authors recommended that in future studies, researchers should design exercise interventions using a functionally based approach to ensure that every participant receives a similar exercise stimulus.

In addition to addressing the physiologic training needs in people with physical disabilities, it is also important to ensure that the interventions are accessible. Common barriers to exercise experienced by people with physical disabilities include lack of transportation to and from exercise venues, cost of the program, inaccessible fitness facilities, and time constraints [5]. Fortunately, there are a growing number of scalable telehealth technologies that have the potential to reach large numbers of people with disabilities in the comfort of their home [6]. In a recent review, we found that technology-driven exercise intervention studies sustained 56% of all outcomes measured at follow-up, compared to 24% for the exercise intervention studies that used technology only for measurement purposes [7]. Given the low rate of exercise participation among people with physical disabilities [8], there is a pressing need to continue exploring the potential of using telehealth technologies to deliver home-based exercise, or what we refer to as teleexercise, among people with disabilities.

Providing exercise that is enjoyable is also important as it will likely increase adherence to the exercise program in people with physical disabilities. Many fitness-related training protocols such as treadmill walking, stationary cycling, and weight lifting often have low adherence rates due to lack of enjoyment or social interaction [9–11]. Asano et al. surveyed 417 people with multiple sclerosis and found “dislike exercise” and “find exercise boring” were common reasons for not exercising [12]. Providing alternative and enjoyable forms of exercise thus becomes critical in helping individuals find activities that fit their interests and needs. Several studies have reported that music is an effective strategy for improving exercise adherence [13–16] and enjoyment [17, 18]. Listening to music during exercise is associated with positive moods and feelings [19]. Indeed, evidence suggests that dance is a promising intervention for improving physical function in people with Parkinson’s [20, 21], stroke [22], and other disabling conditions [23–25]. An additional benefit is that movement-based exercise with music can be performed almost anywhere with minimal equipment.

### Objectives {7}

The movement-to-music (M2M) study aims to examine a rhythmic-based teleexercise intervention (eM2M) with 108 adults with physical disabilities. Specifically, the **primary aim** is to investigate effects of the 12-week eM2M intervention on physical and psychosocial health outcomes in participants who are classified into three functional mobility groups: Group I—only able to exercise while sitting, Group II—able to exercise sitting and standing with or without support, and Group III—able to exercise one side of the body more than the other side. We hypothesize that participants who receive the 12-week eM2M intervention will have the following:

1. Significantly higher gains in cardiorespiratory fitness and muscle strength compared to waitlist control (*WC*) participants post-intervention.
2. Significantly greater gains in lower extremity function, physical activity, health-related quality of life, and social participation compared to *WC* participants post-intervention.

The **secondary aim** is to compare the effect sizes of eM2M on physical health outcomes including cardiorespiratory fitness, muscle strength, and lower extremity function with those observed in a previous M2M trial [26] that grouped participants based on disability type. We hypothesize that gains in physical health will be larger than in the previous M2M trial where participants were grouped by disability type.

The **tertiary aim** is to explore the role of group cohesion, instructor support, exercise self-efficacy, exercise self-regulation, social support for exercise, and outcome expectations for exercise as mediating variables between eM2M and the primary and secondary outcomes. The heterogeneity of treatment effect across the physical health outcomes will also be examined using functional mobility and disability type as moderators.

### Trial design {8}

**Fig. 1:**
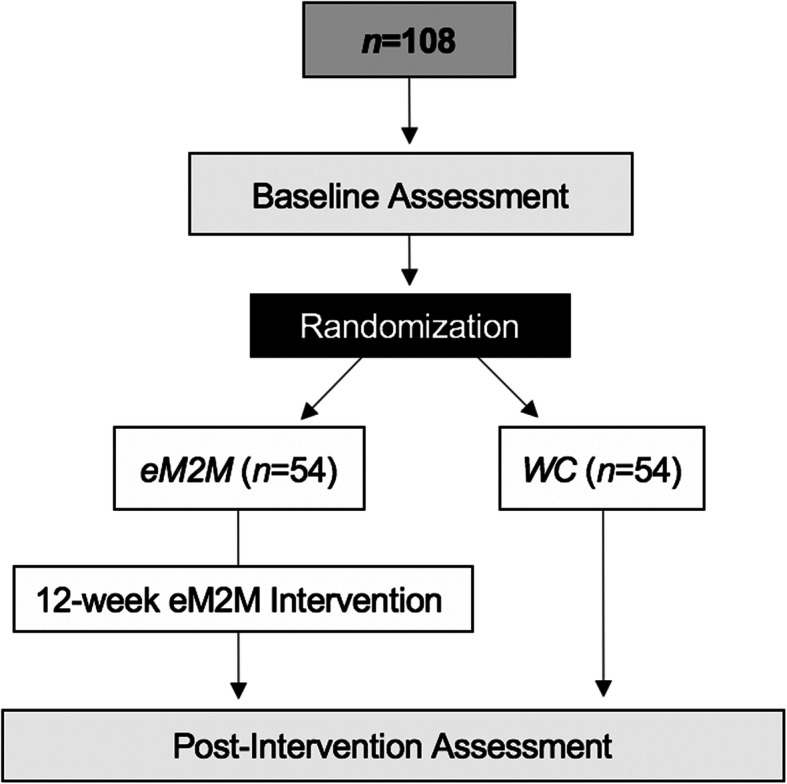
Overall M2M study structure

The M2M study is a two-arm randomized controlled efficacy trial. Eligible and enrolled participants will be randomized into one of two study arms: *eM2M* and *WC*. The overall study structure is illustrated in Fig. 1.

## Methods: participants, interventions, and outcomes

### Study setting {9}

The entire study will be conducted remotely through a telehealth facility located at the study site. All physical outcomes will be assessed via a HIPAA-protected Zoom videoconferencing platform (teleassessments). All self-report outcomes will be collected using electronic surveys delivered through the Research Electronic Data Capture (REDCap). The eM2M intervention will be delivered through the same Zoom platform, which is connected to a custom built, password-protected intervention website housed within an encrypted server at the university. The website will be used to collect vital signs and physical condition questions before and after each eM2M session.

### Eligibility criteria [27]

Individuals are eligible to participate in the study after meeting all inclusion criteria, which include (1) primary diagnosis of traumatic brain injury, stroke, multiple sclerosis, spinal cord injury, spina bifida, Parkinson’s disease, or cerebral palsy conferred by a physician; (2) fits one of the three functional mobility groups: Groups I–III; (3) between the ages of 18 and 70 years; (4) physician clearance to participate; (5) willing to participate in an exercise program 3 times per week; (6) conversant in and reads English; (7) have internet access. The exclusion criteria are (1) participated in a short-term rehabilitation program or a similar intervention in the last 6 months; (2) use of tobacco products in the last 6 months; (3) score <19 on the Telephone Interview for Cognitive Status [27]; (4) active pressure ulcer; (5) any contraindications to exercise based on the American College of Sports Medicine (ACSM) guidelines [28]; (6) visual acuity that prevents following a group exercise class; (7) significant hearing impairment impeding ability to hear music to engage in exercise; and (8) currently pregnant.

### Who will take informed consent? {26a}

Upon completion of the screening process with a study recruitment coordinator, eligible individuals will receive an electronic consent form via REDCap and will officially enroll in the study after signing the informed consent form.

### Additional consent provisions for the collection and use of participant data and biological specimens {26b}

This study does not involve collecting biological specimens for storage. The consent form asks if participants are willing to be contacted for future research opportunities. Participants can decline to this option and still participate in the M2M study.

### Interventions

Enrolled participants who complete baseline assessments will be randomly assigned to *eM2M* or *WC*. After randomization, participants in the *eM2M* arm will meet virtually with study staff who will provide information about the intervention schedule and an online program (e-STORIES) [29] on exercise self-regulation strategies, including goal setting, to be completed prior to beginning the intervention. Participants in *WC* will be instructed to maintain their usual activities during the 12-week intervention and will be invited to participate in an eM2M program after the control period. Both *eM2M* and *WC* participants will be asked to complete a weekly electronic survey on their physical activity level.

### Explanation for the choice of comparators {6b}

The eM2M intervention is designed to progressively increase exercise duration to meet the U.S. recommended exercise guidelines of 150 min of moderate-intensity physical activity every week [30]. The intervention utilizes movement routines choreographed to music to target four fitness components: range of motion, muscular strength, cardiorespiratory endurance, and balance. Table 1 highlights the general structure of the eM2M intervention. The study aims to examine the effects of the 12-week eM2M intervention by comparing *eM2M* participants with *WC* participants.

**Table 1:** eM2M intervention structure

Training component\week		1	2	3	4	5	6	7	8	9	10	11	12
		Duration (minute)											
**Warm up** **(Range of motion)**	**Upper body**	5	5	5	5	5	5	5	5	5	5	5	5
	**Lower body**	5	5	5	5	5	5	5	5	5	5	5	5
**Upper extremity muscle strength**		5	5	5	5	5	5	5	5	5	5	5	5
**Cardiorespiratory fitness**		10	10	15	15	20	20	25	25	30	30	30	30
**Lower extremity muscle strength**		5	5	5	5	5	5	0	0	0	0	0	0
**Balance**	**Static**	5	3	3	2	2	0	0	0	0	0	0	0
	**Dynamic**	0	2	2	3	3	5	5	5	0	0	0	0
**Cool down/mindfulness**		5	5	5	5	5	5	5	5	5	5	5	5
**Total minutes**		**40**	**40**	**45**	**45**	**50**	**50**	**50**	**50**	**50**	**50**	**50**	**50**

### Intervention description {11a}

The eM2M intervention consists of three sessions per week for a total of 36 sessions over 12 weeks. The intervention provides three different programs based upon participants’ functional mobility, which are classified into: Group I (seated exercise only), Group II (seated and standing exercise with or without support while standing), and Group III (exercise for right and left side of the body).

#### Utilization of the Zoom platform and technology

All participants will be mailed a laptop after they officially enroll in the study. The laptop will be used for completing the teleassessments with study staff as well as participating in the intervention. The laptop will have a preinstalled shortcut on its desktop for easy access to the eM2M sessions. The shortcut will direct *eM2M* participants to the password-protected intervention website that will prompt them to input vital signs and answer questions on rating of perceived exertion (RPE), pain, and fatigue when they login 15 min prior to each eM2M session. The vital signs include resting blood pressure, heart rate, and peripheral oxygen saturation that are measured with a portable blood pressure monitor and a pulse oximeter mailed with the laptop. If participants’ vital signs are within safe range for exercise (blood pressure ≤ 180/110 mmHg; heart rate ≤ 100 bpm; oxygen saturation ≥ 90%), participants will then be directed to the Zoom platform for the eM2M session. If a vital sign is not within a safe range, participants will be redirected to a webpage that informs them to rest for a few minutes before attemping to login again. If upon a second attempt a vital sign is still not within a safe range, participants will be instructed to call study staff and be recommended to not join the class that day.

All eM2M sessions will be delivered by a trained M2M instructor from the Telehealth Center at Lakeshore Foundation, Birmingham, AL. The sessions will be supported by a research assistant, who will facilitate the instructor and participants with technical difficulties and ensure delivery quality of the eM2M sessions. At the beginning of each session, the instructor will ask all participants to set their Zoom screens to gallery view so everyone can talk and interact with each other. Before starting the first movement routine, the research assistant will spotlight the instructor’s video to ensure participants are able to easily view and follow the instructor’s movements. The research assistant will also mute participants’ microphones to prevent background noise from interfereing with the session. Participants will be instructed to raise their hand if they encounter any issues during the session, and the research assistant will transfer the participants to a Zoom breakout room to resolve the issues. Toward the end of the session, the instructor will ask all participants to change back to gallery view for a cool-down and mindfulness routine so everyone can see each other in one screen. Once the session concludes the intervention website will redirect participants to input their vital signs and complete the same questions that they completed before the session.

#### Components of the eM2M intervention

The eM2M intervention is highly structured to ensure strong fidelity between instructors. Each session consist of 7 to 8 movement routines accompanied with music. Every routine incorporates combinations of various movement patterns and sequences that target one of the four fitness components described below and can be adapted to the participants’ functional mobility level.

#### Range of motion component

Each session starts with two 5-min range of motion routines that serve as the warm-up and focus on upper and lower extremity joint movements performed in a seated position. The upper range of motion routine targets upper limb movements including (1) neck flexion, extension, lateral flexion, and rotation; (2) shoulder extension, lateral/frontal/dorsal extension, flexion, adduction, horizontal adduction, abduction, horizontal abduction, circumduction, and rotation; (3) shoulder girdle elevation and depression; (4) elbow extension and flexion; (5) wrist extension, flexion, and rotation; (6) hand extension and flexion; and (7) torso lateral/frontal/dorsal extension and rotation. The lower range of motion routine targets lower limb movements including (1) hip extension, flexion, adduction, horizontal adduction, abduction, horizontal abduction, and rotation; (2) knee extension and flexion; (3) ankle extension, flexion, and rotation; and (4) toes extension and flexion. The movement tempo ranges from 12 to 60 beats per minute (bpm).

#### Muscle strengthening component

The muscle strengthening component consists of one 5-min seated routine that targets upper extremity muscle groups including biceps, triceps, deltoid, trapezius/rhomboids, pectorals, erector spinae, rectus abdominus, and obliques as well as one 5-min standing routine that target lower extremity muscle groups including quadriceps, hamstrings, gastrocnemius/soleus, and tibialis anterior. The routines are adapted for Group I participants, where the upper extremity strength routine focuses on the shoulder, chest, upper back, and arm muscles and the lower extremity strength routine focuses on the abdominal and lower back muscles. All *eM2M* participants receive a pair of 2-lb wrist weights mailed to them after randomization that are used to perform this routine. The movement tempo ranges from 40 to 60 bpm.

#### Cardiorespiratory fitness component

The cardiorespiratory fitness component consists of two routines that are performed at movement tempo that ranges from 100 to 180 bpm. The routines are structured with a series of movement combinations and can be performed seated or standing with or without support. The component begins with 10 min at week 1, progress to 30 min at week 9, and maintain 30 min for remaining weeks 10–12. Participants are asked to rate their RPE on a 0–10 Borg scale [31, 32] at the end of each cardiorespiratory fitness routine.

#### Balance component

The balance component consists of a 5-min routine that targets static and dynamic balance. This component begins with a static balance routine at week 1 and progresses to a dynamic balance-focused routine at week 8. The routine is adapted for Group I participants, which involves sitting stability, trunk rotation, and weight shifting with arms and upper body reaching in different directions.

#### Cool down/mindfulness component

The cool down/mindfulness component focuses on breathing and mindfulness where the instructor and participants come together while forming a virtual, imaginary circle. The instructor then delivers a mindfulness quote and ask participants to share their thoughts at the end of each session.

#### *Participant safety*

Before starting the intervention, study staff will confirm with the participants about their emergency contact information and instruct them to notify their emergency contacts about the intervention schedule. In the event of an emergency during the eM2M session, the research assistant will place the participant in a breakout room and confirm severity of the emergency. Should the emergency deem that medical help is necessary, study staff will first contact the participant’s emergency contact and will call 911 if the emergency contact cannot be reached.

### Criteria for discontinuing or modifying allocated interventions {11b}

There are no criteria for discontinuing or modifying allocated Intervention when requested by participants. Participants may choose to stop the intervention or withdraw from the study for any reason, including being assigned to a study arm that is not a preference.

### Strategies to improve adherence to interventions {11c}

The eM2M intervention is underpinned by four Social Cognitive Theory constructs: self-efficacy, self-regulation, outcome expectations, and social support [33]. Self-efficacy is targeted through (1) *mastery experience*: begin with lower exercise intensity and movement complexity and progress to higher exercise intensity and movement complexity across the intervention, to ensure that participants are challenged in a progressive manner; (2) *verbal persuasion*: motivate and encourage participants during class through verbal positive reinforcement; and (3) *emotional/physiological states*: ask participants to rate their RPE as well as pain and fatigue level at the beginning and end of each eM2M session to increase awareness of emotional and physiological states. Self-regulation and outcome expectations are addressed by asking participants to set monthly exercise goals and discuss expectations they have about exercise. In addition, the eM2M instructors will explain targeted outcomes to participants at the beginning of each routine. Social support is addressed by having group classes where participants with similar functional mobility levels are exercising together and by giving participants time at the beginning and end of each class to talk and interact with each other. The purpose of having gallery view on the Zoom platform at the beginning and end of each session is to facilitate the group interaction and social support. In addition, email or text reminders will be sent to participants a day before the intervention starts and one hour prior to each eM2M session.

### Relevant concomitant care permitted or prohibited during the trial {11d}

Not applicable, no concomitant care or interventions are prohibited during the trial.

### Provisions for post-trial care {30}

Not applicable, no provisions for post-trial care are included in the study.

### Outcomes {12}

Primary and secondary outcomes include a set of physical assessments and self-report questionnaires and will be assessed at baseline and post 12-week intervention via the Zoom platform (teleassessments [34]) and REDCap electronic questionnaires. Assessors will be blinded to participant assignment, and allocation of the arm assignment will not be done until baseline testing is completed. The outcome measures included in this study were developed and validated in previous literature with adequate to excellent reliability. The primary outcomes include changes in cardiorespiratory fitness measured with resting heart rate [35] and the heart rate recovery test [36, 37] as well as muscle strength assessed with hand-held dynamometer [38–40] at post 12-week intervention. The secondary outcomes include changes in lower extremity function assessed with the Short Physical Performance Battery [41] and the Timed Up and Go tests [42–44], health-related quality of life measured using the NIH PROMIS 10 Global Health Items [45], social participation measured using the NIH PROMIS Ability to Participate in Social Roles and Activities Short Form 8a [45], and physical activity measured using the Godin Leisure Time Exercise Questionnaire [46–54] at post 12-week intervention. Table 2 summarizes all outcome measures and the corresponding data collection time points.

**Table 2:** Outcome measures of the M2M study

Role	Variables	Outcome measures	Collection time points
**Primary outcomes**	Cardiorespiratory fitness	Resting heart rate [35] Heart rate recovery test [36, 37]	Baseline and 12 weeks
	Muscle strength	Hand-held dynamometer [38–40]	Baseline and 12 weeks
**Secondary outcomes**	Lower extremity function	Short physical performance battery [41] Timed up and go [42–44]	Baseline and 12 weeks
	Health-related quality of life	NIH PROMIS 10 Global Health Items [45]	Baseline and 12 weeks
	Social participation	NIH PROMIS Ability to Participate in Social Roles and Activities Short Form 8a [45]	Baseline and 12 weeks
	Physical activity	Godin Leisure Time Exercise Questionnaire [46–54]	Baseline and 12 weeks
**Mediators**	Program adherence	Percentage of # of attended eM2M sessions	12-week intervention period
	Group cohesion	Physical Activity Group Environment Questionnaire [55–57]	12 weeks
	Instructor support	Perceived Autonomy Support Scale for Exercise Settings [57, 58]	12 weeks
	Exercise self-efficacy	Exercise Self-efficacy Scale [59]	Baseline and 12 weeks
	Exercise self-regulation	Exercise Goal-setting Scale [60]	Baseline and 12 weeks
	Outcome expectations for exercise	Multidimensional Outcome Expectations for Exercise Scale [61]	Baseline and 12 weeks
	Social support for exercise	Social Provisions Scale [62]	Baseline and 12 weeks
**Moderators**	Functional mobility	Short Physical Performance Battery [41]	Baseline
	Disability type	Demographics and Health History Questionnaire	Baseline
**Covariates**	Barriers in physical activity	Barriers in Physical Activity Questionnaire [63]	Baseline

In addition, after participants complete the eM2M intervention, they will be asked to participate in semi-structured interviews via Zoom. The interviews will be conducted in a group format (the same group of participants that underwent the intervention together). The interviews will include questions that probe participants’ perceived impact of eM2M on their health and function, as well as their experience exercising with other participants with similar functional levels who have different disabilities. The interviews will be audio-recorded, transcribed, and analyzed using a latent thematic analysis approach (underpinned by interpretivism).

### Participant timeline {13}

Table 3 displays the schedule of enrollment, randomization, intervention, and assessments for the M2M study participants.

**Table 3:**
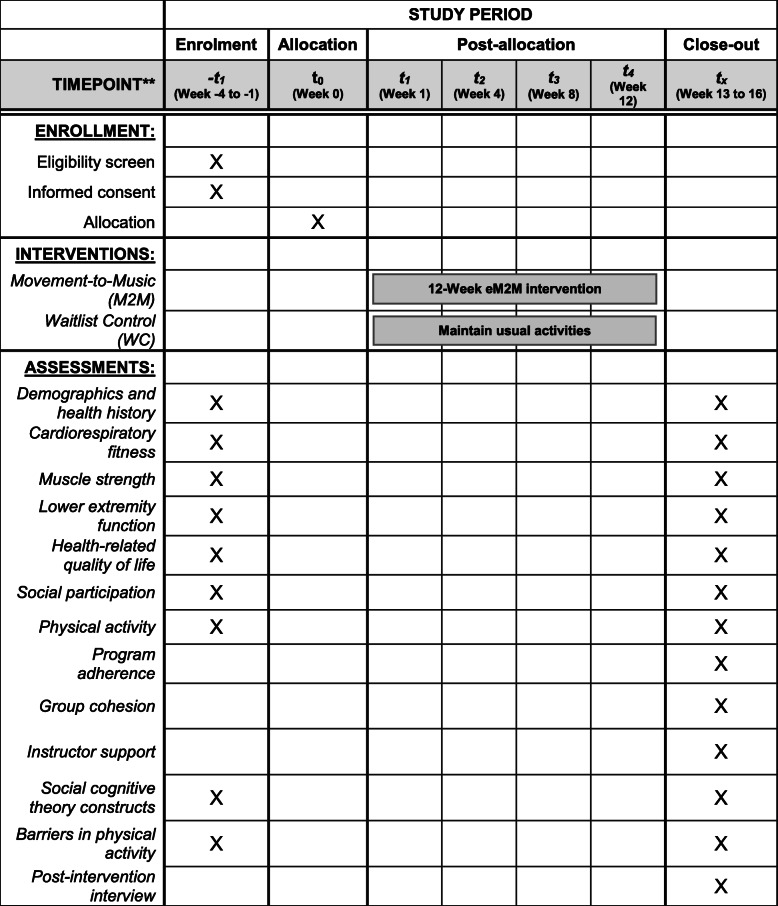
Schedule of enrollment, randomization, intervention, and assessments for the M2M study participants

### Sample size {14}

Our conservative sample size and power calculations for the primary outcomes, which constitute a family of hypotheses [63], assume a two-sided overall family-wise error rate of 0.05 [64], 80% power, an analysis of covariance (ANCOVA)-based approach, and an attrition rate of 25% with 82 participants finishing the study. Under these assumptions, we will have 80% power to detect at least a minimally detectable difference (Cohen’s *d*) of 0.552. In these calculations, a conservative correlation of 0.6 between the baseline covariate and outcome is assumed, which is similar to a previous study by Froehlich-Grobe et al. [4].

### Recruitment {15}

Potential participants will be primarily reached by disseminating recruitment information through local disability service providers, their social media outlets, and the study website (https://m2mstudy.org). If potential participants are interested in participating, they will be directed to the study website to complete an electronic pre-screening form linked to REDCap.

### Assignment of interventions: allocation

#### Sequence generation {16a}

Randomization is stratified based on the three functional mobility groups. Within each stratum of 36 participants, a permuted block randomization design is implemented to ensure close balance between the two arms across intervention waves (12 weeks per wave) and to increase the unpredictability in the upcoming assignment and prevent inadvertent bias. The randomization sequence was generated by a study biostatistician (TM) who is not involved in participant recruitment, assessments, and intervention delivery.

#### Concealment mechanism {16b}

The randomization sequence is uploaded to a randomization module in REDCap.

#### Implementation {16c}

Randomization will be performed entirely in REDCap by study staff after a participant completes baseline assessment.

### Assignment of interventions: blinding

#### Who will be blinded {17a}

Given the nature of the intervention, it is not plausible to blind participants and study staff who perform the randomization and monitor the intervention delivery. However, all data collection and analyses will be performed by study investigators and staff who are blinded to the study assignments.

#### Procedure for unblinding if needed {17b}

Study personnel who are responsible for collecting and analyzing data will remain blinded throughout the study period.

### Data collection and management

#### Plans for assessment and collection of outcomes {18a}

Before conducting the baseline teleassessments, study staff will connect with each participant for a virtual home visit via Zoom using the laptop mailed to them. The purpose of this visit is to walk participants through all provided testing equipment and identify places at home to exercise safely. The testing equipment includes a blood pressure monitor for measuring resting blood pressure and heart rate, a pulse oximeter for the heart rate recovery test, a hand dynamometer for the grip strength test, and two cones and a tape measure for the Short Physical Performance Battery and the Timed Up and Go tests.

All physical outcomes will be performed by assessors who are trained to administer the teleassessments via Zoom based on standardized protocols. All data from the teleassessments will be entered directly into REDCap. All self-report outcomes will be collected through electronic questionnaire packets delivered via REDCap. All items of the questionnaire packets are set as must-provide-value so participants are required to answer before submission to prevent data missingness. Each questionnaire packet is also set to be delivered to participants three times, separated by 3 days, if it is not completed. A follow-up phone call will be made if participants do not complete the packet after 7 days to ensure participants receive the packet and remind them to complete it within the next 3 days. Randomization will not take place until the the baseline teleassessments and questionnaire packet are completed.

#### Plans to promote participant retention and complete follow-up {18b}

Financial incentives will be provided to participants for completing study assessments, which include $25 for baseline and $50 for post 12-week intervention.

#### Data management {19}

All data will be collected remotely and will be entered directly and stored in REDCap. REDCap is a software program that was developed by Vanderbilt University, with collaboration from a consortium of institutional partners and the NIH National Center for Research Resources, for electronic collection and management of research and clinical trial data. All collected data will be systematically cleaned and verified. Data checking and cleaning involves secondary checks to identify any impossible values (e.g., 0 s to complete Timed Up and Go test) and univariate or multivariate outliers. Any erroneous data points will be corrected and the correction will be documented. REDCap data collection projects rely on a thorough study-specific data dictionary defined in an iterative self-documenting process by all members of the study team.

#### Confidentiality {27}

REDCap is 21 CRF Part 11 capable. Currently, REDCap installations support electronic signatures by positively identifying the user through a unique username and password combination. Access to the REDCap database will be given to the study personnel only, including the biostatistician and data management personnel. Data being analyzed will be exported in de-identified format. As part of the data dictionary development process, individual fields are denoted as “identifiers.” When exporting a de-identified dataset, these variables are omitted. Identities of participants will not be revealed in the presentation or publication of any result from this study. All study personnel are educated about the importance of strictly protecting participants’ rights to confidentiality and can only access the data necessary for job completion. Participants will be informed of law-mandated instances in which confidentially could be breached.

#### Plans for collection, laboratory evaluation, and storage of biological specimens for genetic or molecular analysis in this trial/future use {33}

Not applicable, no biological specimens will be collected in the study.

## Statistical methods

### Statistical methods for primary and secondary outcomes {20a}

#### Primary analysis

The primary analysis for the average treatment effect (**primary aim**) will employ an intent-to-treat linear mixed model ANCOVA with the baseline outcome measures as covariates, the corresponding post-intervention outcome measures as the dependent variables, the intervention as the main factor, and the intervention waves as random effect. In general, missingness will be handled by our choice of mixed models to handle the multi-level data. Mixed models use all the data available to directly estimate model parameters via maximum likelihood or restricted maximum likelihood. Where necessary, we will supplement these primary analyses by using multiple imputation procedures with 10 imputations [4]. We have configured three families of hypotheses based on our primary and secondary outcomes.

For the **secondary aim**, we will estimate the effect sizes and their 95% confidence intervals and compare them with the corresponding effect sizes and interval estimates from a previous M2M trial [65]. We will generate 95% percentile bootstrap confidence intervals between the differences in the effect sizes utilizing data from both trials. To test for pre-specified moderators, we will extend our models in the primary aim to include moderators as main effects along with an interaction term with the intervention. A test for interaction between the moderator and intervention will be conducted.

#### Secondary analysis

Based on the *p* values for all the analyses reported, we will report the false discovery rate and false discovery proportion. We will estimate Bayes factor for each comparison, which is especially useful in the interpretation if the null is not rejected. Using the outcome data, we will conduct exploratory cluster analysis to assess whether the clustering occurs based on functional level or disability type. Descriptive comparisons of *e*M2M effects between disability groups will be reported where appropriate. We will examine barriers in physical activity as well as the four Social Cognitive Theory constructs: self-efficacy, goal-setting, outcome expectancies, and social support as mediating variables between the intervention and the outcomes using the product of coefficients test as suggested by Mackinnon and colleagues [26].

### Interim analyses {21b}

Not applicable, no interim analyses are planned.

### Methods for additional analyses (e.g., subgroup analyses) {20b}

Not applicable, no additional subgroup analyses are planned.

### Methods in analysis to handle protocol non-adherence and any statistical methods to handle missing data {20c}

Missing data will be imputed using multiple imputation where necessary, with assumption that the missingness mechanism is missing at random.

### Plans to give access to the full protocol, participant-level data, and statistical code {31c}

This paper provides the overall study protocol. Readers should contact the authors if interested in other data or documentation of the study.

### Oversight and monitoring

#### Composition of the coordinating centre and trial steering committee {5d}

The study is a single-center trial. All study activities are performed and coordinated under the UAB/Lakeshore Research Collaborative. The day to day support for the study is provided by the following:

1. Scientific director: supervise the implementation and fidelity of the study protocol and intervention delivery.
2. Project coorindator: coordinate all apsects of the study and manage its regulatory requirements.
3. Rercuiment coordinator: recruit, screen, and obtain medical clearance for potential participants and support participant communication throughout the study period.
4. Research assistants: perform day to day study tasks including study visit scheduling, equipment shipping, intervention preparation and monitoring, and participant communication.
5. Study physician: review and approve medical clearance prior to participant enrollment as well as review adverse events.

The scientific director, the project coordinator, the recruitment coordinator, and research assistants communicate about the study on a daily basis. The project coordinator, recruitment coordinator, and research assistants meet weekly to go over project activities and study milestones.

#### Composition of the data monitoring committee, its role, and reporting structure {21a}

The study does not have a Data Monitoring Committee. The study investigator and scientific director (H-JY) will be responsible for monitoring the protocol fidelity, data collection, and data management processes throughout the study period. The study biostatistician (TM) will be responsible for overseeing data analysis and data sharing. The principal investigator (JR), along with all investigators, will be responsible for reporting and preparation of peer-reviewed manuscripts.

#### Adverse event reporting and harms {22}

The M2M study will monitor adverse events and report them based on four types defined by the Behavior Change Consortium of the National Institutes of Health [66–68]. The four types of AEs are (1) falls, (2) cardiovascular-related episodes, (3) musculoskeletal-related events, and (4) health care use. All adverse events will be assessed for severity and causality and will be reported to the Institutional Review Board and relevant regulatory bodies when necessary.

#### Frequency and plans for auditing trial conduct {23}

Not applicable, the study does not contain plans for auditing trial conduct.

#### Plans for communicating important protocol amendments to relevant parties (e.g., trial participants, ethical committees) {25}

The University of Alabama at Birmingham’s Institutional Review Board will be notified when deviations from the approved protocol occur, and the deviations will be fully documented using a problem report form. When important protocol amendments are necessary, the study team will notify and communicate with the program officer of the funding agency. Approvals from the program officer will be obtained prior to amending the protocols. Any amended protocols will be updated in the study’s ClinicalTrials.gov record (https://clinicaltrials.gov/ct2/show/NCT03797378).

#### Dissemination plans {31a}

Findings from this study will be shared publicly and disseminated mainly by publications in peer-reviewed journals as well as conference presentations.

## Discussion

Nearly one out of two people with disabilities (46%) in the USA is categorized as physically inactive [69]. In a 2014 Centers for Disease Control and Prevention report [70], less than 10% of people with physical disabilities performed aerobic exercise, one of the key markers for optimal health and prevention of chronic disease. The report noted that sedentary people with disabilities were three times more likely to report primary health conditions such as heart disease, stroke, diabetes, or cancer compared to active people with disabilities. People with disabilities need immediate resources that they can use to obtain regular exercise.

Being aware of the multiple barriers that many people with disabilities experience when trying to become physically active, there are important questions that this study will address. First, there are very few non-conventional exercise options for people with disabilities. The current study will examine a novel rhythmic exercise intervention that utilizes music to facilitate exercise and will provide evidence on efficacy of this alternative exercise programming. Second, the *e*M2M intervention is designed to include participants with different disabilities and similar functional mobility in a group class setting. The study will test whether a greater intervention effect can be obtained with participants with a more homogeneous range of physical function and will enhance our undestanding of effective design strategies of a home-based exercise intervention for people with disabilities. Third, many people with physical disabilities have difficulty getting to an exercise facility on a regular basis. This is due to factors including but not limited to long delays for transportation services and inaccessible exercise facilities. The M2M study is designed to be delivered completely online so that participants can complete the assessments and intervention in the comfort of their own home. Thus, the study will provide detailed documentation of such an intervention delivery mechanism and will be helpful for designing future studies involving people with disabilities.

### Trial status

Protocol Version 1, Novemeber 2020. Recruitment started in December 2020 and the first intervention wave is projected to take place in March 2021. Recruitment is expected to be completed in July 2022.

## Abbreviations

M2M: Movement-to-music
eM2M: Remotely delivered movement-to-music intervention
WC: Waistlist control
REDCap: Research electronic data capture
RPE: Rating of perceived exertion
PROMIS: Patient-Reported Outcomes Measurement Information System
ANCOVA: Analysis of covariance

## References

[1] Rimmer JH, Chen M-D, McCubbin JA, Drum C, Peterson J (2010). Exercise intervention research on persons with disabilities: what we know and where we need to go. Am J Phys Med Rehabil..

[2] Lai B, Young H-J, Bickel CS, Motl RW, Rimmer JH (2017). Current trends in exercise intervention research, technology, and behavioral change strategies for people with disabilities. Am J Phys Med Rehabil..

[3] Lai B, Cederberg K, Vanderbom KA, Bickel CS, Rimmer JH, Motl RW (2018). Characteristics of adults with neurologic disability recruited for exercise trials: a secondary analysis. Adapt Phys Activ Q..

[4] Froehlich-Grobe K, White GW (2004). Promoting physical activity among women with mobility impairments: a randomized controlled trial to assess a home- and community-based intervention 11No commercial party having a direct financial interest in the results of the research supporting this article has or will confer a benefit upon the author(s) or upon any organization with which the author(s) is/are associated. Arch Phys Med Rehabil..

[5] Rimmer JH, Riley B, Wang E, Rauworth A, Jurkowski J (2004). Physical activity participation among persons with disabilities: barriers and facilitators. Am J Prev Med..

[6] Rimmer J, Lai B, Young H-J (2016). Bending the arc of exercise and recreation technology toward people with disabilities. Arch Phys Med Rehabil..

[7] Lai B, Kim Y, Wilroy J, Bickel CS, Rimmer JH, Motl RW (2019). Sustainability of exercise intervention outcomes among people with disabilities: a secondary review. Disabil Rehabil..

[8] Hollis N, Zhang QC, Cyrus AC, Courtney-Long E, Watson K, Carroll DD (2020). Physical activity types among US adults with mobility disability, Behavioral Risk Factor Surveillance System, 2017. Disabil Health J..

[9] Pridgeon L, Grogan S (2012). Understanding exercise adherence and dropout: an interpretative phenomenological analysis of men and women’s accounts of gym attendance and non-attendance. Qual Res Sport Exerc Health..

[10] Resnick B, Spellbring AM (2000). Understanding what motivates older adults to exercise. J Gerontol Nurs..

[11] Huberty JL, Ransdell LB, Sidman C, Flohr JA, Shultz B, Grosshans O, Durrant L (2008). Explaining long-term exercise adherence in women who complete a structured exercise program. Res Q Exerc Sport..

[12] Asano M, Duquette P, Andersen R, Lapierre Y, Mayo NE (2013). Exercise barriers and preferences among women and men with multiple sclerosis. Disabil Rehabil..

[13] Annesi JJ (2001). Effects of music, television, and a combination entertainment system on distraction, exercise adherence, and physical output in adults. Can J Behav Sci..

[14] Karageorghis CI, Terry PC, Lane AM (1999). Development and initial validation of an instrument to assess the motivational qualities of music in exercise and sport: the Brunel Music Rating Inventory. J Sports Sci..

[15] Miller T, Swank A, Manire J, Robertson R, Wheeler B (2010). Effect of music and dialog on perception of exertion, enjoyment, and metabolic responses during exercise. Int J Fit..

[16] Schwartz SE, Fernhall B, Plowman SA (1990). Effects of music on exercise performance. J Cardiopulm Rehabil Prev..

[17] Wininger SR, Pargman D (2003). Assessment of factors associated with exercise enjoyment. J Music Ther..

[18] Shaulov N, Lufi D (2009). Music and light during indoor cycling. Percept Mot Skills..

[19] Karageorghis CI, Priest DL (2012). Music in the exercise domain: a review and synthesis (Part II). Int Rev Sport Exerc Psychol..

[20] Hackney ME, Earhart GM (2010). Recommendations for implementing tango classes for persons with Parkinson disease. Am J Dance Ther..

[21] Hackney ME, Earhart GM (2010). Effects of dance on gait and balance in Parkinson’s disease: a comparison of partnered and nonpartnered dance movement. Neurorehabil Neural Repair..

[22] Hackney M, Hall C, Echt K, Wolf S (2012). Application of adapted tango as therapeutic intervention for patient with chronic stroke. J Geriatr Phys Ther..

[23] Hokkanen L, Rantala L, Remes AM, Härkönen B, Viramo P, Winblad I (2008). Dance and movement therapeutic methods in management of dementia: a ramdomized, controlled study. J Am Geriatr Soc..

[24] Abreu M, Hartley G (2013). The effects of salsa dance on balance, gait, and fall risk in a sedentary patient with Alzheimer’s dementia, multiple comorbidities, and recurrent falls. J Geriatr Phys Ther..

[25] Hackney ME, Earhart GM (2009). Effects of dance on movement control in parkinson’s disease: a comparison of argentine tango and American ballroom. Journal of rehabilitation medicine. J Rehabil Med..

[26] Young H-J, Mehta TS, Herman C, Wang F, Rimmer JH (2019). The effects of M2M and adapted yoga on physical and psychosocial outcomes in people with multiple sclerosis. Arch Phys Med Rehabil..

[27] Brandt J, Specter M, Folstein MF (1988). The telephone interview for cognitive status. Neuropsychiatry Neuropsychol Nehavioral Neurol.

[28] Ferguson B. ACSM’s guidelines for exercise testing and prescription. 9th Ed. 2014. J Canadian Chiropr Assoc. 58(3):328.

[29] Wilroy JD, Martin Ginis KA, Rimmer JH, Wen H, Howell J, Lai B (2019). An E-learning program for increasing physical activity associated behaviors among people with spinal cord injury: usability study. JMIR Form Res..

[30] U.S. Department of Health and Human Services. 2008 Physical Activity Guidelines for Americans Washington (DC): U.S. Department of Health and Human Services. 2008. http://www.health.gov/paguidelines. Accessed 20 Oct 2020.

[31] Penko AL, Barkley JE, Koop MM, Alberts JL (2017). Borg scale is valid for ratings of perceived exertion for individuals with Parkinson’s disease. Int J Exerc Sci..

[32] Borg GA (1982). Psychophysical bases of perceived exertion. Med Sci Sports Exerc..

[33] Ma JK, Martin Ginis KA (2018). A meta-analysis of physical activity interventions in people with physical disabilities: content, characteristics, and effects on behaviour. Psychol Sport Exerc..

[34] Lai B, Chiu CY, Pounds E, Tracy T, Mehta T, Young HJ, Riser E, Rimmer J (2020). A description of Covid-19 modifications to the TEAMS study protocol for remotely delivering teleassessment/teletraining of complementary alternative medicine among people with multiple sclerosis: protocol for a randomized controlled effectiveness trial. JMIR Res Protoc..

[35] Saxena A, Minton D, Lee D-C, Sui X, Fayad R, Lavie CJ (2013). Protective role of resting heart rate on all-cause and cardiovascular disease mortality. Mayo Clin Proc.

[36] Sydó N, Sydó T, Gonzalez Carta KA, Hussain N, Farooq S, Murphy JG, Merkely B, Lopez-Jimenez F, Allison TG (2018). Prognostic performance of heart rate recovery on an exercise test in a primary prevention population. J Am Heart Assoc..

[37] Morshedi-Meibodi A, Larson MG, Levy D, O'Donnell CJ, Vasan RS (2002). Heart rate recovery after treadmill exercise testing and risk of cardiovascular disease events (The Framingham Heart Study). Am J Cardiol..

[38] Boissy P, Bourbonnais D, Carlotti MM, Gravel D, Arsenault BA (1999). Maximal grip force in chronic stroke subjects and its relationship to global upper extremity function. Clin Rehabil..

[39] Lindstrom-Hazel D, Kratt A, Bix L (2009). Interrater reliability of students using hand and pinch dynamometers. Am J Occup Ther..

[40] Bertrand AM, Mercier C, Bourbonnais D, Desrosiers J, Gravel D (2007). Reliability of maximal static strength measurements of the arms in subjects with hemiparesis. ClinRehabil..

[41] Guralnik JM, Ferrucci L, Pieper CF, Leveille SG, Markides KS, Ostir GV, Studenski S, Berkman LF, Wallace RB (2000). Lower extremity function and subsequent disability: consistency across studies, predictive models, and value of gait speed alone compared with the short physical performance battery. J Gerontol A Biol Sci Med Sci..

[42] Bennie S, Bruner K, Dizon A, Fritz H, Goodman B, Peterson S (2003). Measurements of balance: comparison of the timed "up and go" test and functional reach test with the berg balance scale. J Phys Ther Sci..

[43] Nilsagard Y, Lundholm C, Gunnarsson L, Dcnison E (2007). Clinical relevance using timed walk tests and ‘timed up and go’ testing in persons with multiple sclerosis. Physiother Res Int..

[44] Learmonth YC, Paul L, McFadyen AK, Mattison P, Miller L (2012). Reliability and clinical significance of mobility and balance assessments in multiple sclerosis. Int J Rehabil Res..

[45] Hays RD, Bjorner JB, Revicki DA, Spritzer KL, Cella D (2009). Development of physical and mental health summary scores from the patient-reported outcomes measurement information system (PROMIS) global items. Qual Life Res..

[46] Motl RW, Bollaert RE, Sandroff BM (2018). Validation of the Godin Leisure-Time Exercise Questionnaire classification coding system using accelerometry in multiple sclerosis. Rehabil Psychol..

[47] Motl RW, McAuley E, Klaren R (2014). Reliability of physical-activity measures over six months in adults with multiple sclerosis: implications for designing behavioral interventions. Behav Med..

[48] Gosney JL, Scott JA, Snook EM, Motl RW (2007). Physical activity and multiple sclerosis: validity of self-report and objective measures. Fam Community Health..

[49] Motl RW, McAuley E, Snook EM, Scott JA (2006). Validity of physical activity measures in ambulatory individuals with multiple sclerosis. Disabil Rehabil..

[50] Kinnett-Hopkins D, Grover SA, Yeh EA, Motl RW (2016). Physical activity in pediatric onset multiple sclerosis: validating a questionnaire for clinical practice and research. Mult Scler Relat Disord..

[51] Weikert M, Motl RW, Suh Y, McAuley E, Wynn D (2010). Accelerometry in persons with multiple sclerosis: measurement of physical activity or walking mobility?. J Neurol Sci..

[52] Snook EM, Motl RW, Gliottoni RC (2009). The effect of walking mobility on the measurement of physical activity using accelerometry in multiple sclerosis. Clin Rehabil..

[53] Weikert M, Suh Y, Lane A, Sandroff B, Dlugonski D, Fernhall B, Motl RW (2012). Accelerometry is associated with walking mobility, not physical activity, in persons with multiple sclerosis. Med Eng Phys..

[54] Sandroff BM, Dlugonski D, Weikert M, Suh Y, Balantrapu S, Motl RW (2012). Physical activity and multiple sclerosis: new insights regarding inactivity. Acta Neurol Scand..

[55] Estabrooks PA, Carron AV (2000). The Physical Activity Group Environment Questionnaire: an instrument for the assessment of cohesion in exercise classes. Group Dyn-Theor Res..

[56] Lee RE, O'Connor DP, Smith-Ray R, Mama SK, Medina AV, Reese-Smith JY, Banda JA, Layne CS, Brosnan M, Cubbin C, McMillan T, Estabrooks PA (2012). Mediating effects of group cohesion on physical activity and diet in women of color: health is power. Am J Health Promot..

[57] Gilbert M, Chaubet P, Karelis A, Dancause KN (2017). Perceptions of group exercise courses and instructors among Quebec adults. BMJ Open Sport Exerc Med..

[58] Hagger MS, Chatzisarantis NLD, Hein V, Pihu M, Soós I, Karsai I (2007). The perceived autonomy support scale for exercise settings (PASSES): development, validity, and cross-cultural invariance in young people. Psychol Sport Exerc..

[59] McAuley E (1993). Self-efficacy and the maintenance of exercise participation in older adults. J Behav Med..

[60] Rovniak LS, Anderson ES, Winett RA, Stephens RS (2002). Social cognitive determinants of physical activity in young adults: a prospective structural equation analysis. Ann Behav Med..

[61] Wójcicki TR, White SM, McAuley E (2009). Assessing outcome expectations in older adults: the multidimensional outcome expectations for exercise scale. J Gerontol B Psychol Sci Soc Sci..

[62] Cutrona CE, Russell DW. The provisions of social relationships and adaptation to stress. In: Jones WH, Perlman D, editors. Advances in Personal Relationships. Greenwich: JAI Press; 1987. p.37-67.

[63] Vasudevan V, Rimmer JH, Kviz F (2015). Development of the barriers to physical activity questionnaire for people with mobility impairments. Disabil Health J..

[64] Shaffer JP (1995). Multiple hypothesis testing. Annul Rev Psychol..

[65] Li P, Stuart EA, Allison DB (2015). Multiple imputation: a flexible tool for handling missing data. JAMA..

[66] Mackinnon DP, Lockwood CM, Williams J (2004). Confidence limits for the indirect effect: distribution of the product and resampling methods. Multivariate Behav Res..

[67] MacKinnon DP, Fairchild AJ, Fritz MS (2007). Mediation analysis. Annu Rev Psychol..

[68] Mackinnon DP (2011). Integrating mediators and moderators in research design. Res Soc Work Pract..

[69] Ory M, Resnick B, Jordan PJ, Coday M, Riebe D, Garber CE, Pruitt L, Bazzarre T (2005). Screening, safety, and adverse events in physical activity interventions: collaborative experiences from the behavior change consortium. Ann Behav Med..

[70] Carroll DD, Courtney-Long EA, Stevens AC, Sloan ML, Lullo C, Visser SN, Fox MH, Armour BS, Campbell VA, Brown DR, Dorn JM, Centers for Disease Control and Prevention (CDC) (2014). Vital signs: disability and physical activity--United States, 2009-2012. MMWR Morb Mortal Wkly Rep..

